# Novel Candidate Key Drivers in the Integrative Network of Genes, MicroRNAs, Methylations, and Copy Number Variations in Squamous Cell Lung Carcinoma

**DOI:** 10.1155/2015/358125

**Published:** 2015-02-23

**Authors:** Tao Huang, Jing Yang, Yu-dong Cai

**Affiliations:** ^1^College of Life Science, Shanghai University, Shanghai 200444, China; ^2^Institute of Health Sciences, Shanghai Institutes for Biological Sciences, Chinese Academy of Sciences and Shanghai Jiao Tong University School of Medicine, Shanghai 200031, China

## Abstract

The mechanisms of lung cancer are highly complex. Not only mRNA gene expression but also microRNAs, DNA methylation, and copy number variation (CNV) play roles in tumorigenesis. It is difficult to incorporate so much information into a single model that can comprehensively reflect all these lung cancer mechanisms. In this study, we analyzed the 129 TCGA (The Cancer Genome Atlas) squamous cell lung carcinoma samples with gene expression, microRNA expression, DNA methylation, and CNV data. First, we used variance inflation factor (VIF) regression to build the whole genome integrative network. Then, we isolated the lung cancer subnetwork by identifying the known lung cancer genes and their direct regulators. This subnetwork was refined by the Bayesian method, and the directed regulations among mRNA genes, microRNAs, methylations, and CNVs were obtained. The novel candidate key drivers in this refined subnetwork, such as the methylation of ARHGDIB and HOXD3, microRNA let-7a and miR-31, and the CNV of AGAP2, were identified and analyzed. On three large public available lung cancer datasets, the key drivers ARHGDIB and HOXD3 demonstrated significant associations with the overall survival of lung cancer patients. Our results provide new insights into lung cancer mechanisms.

## 1. Introduction

Lung cancer is the most common cause of cancer-related death worldwide, and non-small-cell lung cancer (NSCLC) accounts for approximately 80% of all cases [1]. The overall 5-year survival rate remains low despite the development of clinical diagnosis techniques and chemotherapy [2]. NSCLC has two major subtypes: squamous cell lung carcinoma (SCC) and lung adenocarcinoma (AD). SCC represents approximately 20–30% of NSCLC patients and is characterized by keratinization in squamous pearls and the formation of intercellular bridges [3]. Many studies have provided insight into several driver genes, miRNAs, and crucial signaling pathways that contribute to lung cancer pathogenesis.

Genetic and epigenetic alterations are frequently found in SCC. For example, Sriram et al. [4] found that lung squamous cell carcinoma patients with the loss of SOCS6 have worse disease-free and overall survival rates. Son et al. [5] detected gains at 1p31.1, 3q26.1, and 3q26.31–3q29 and losses at 1p21.1, 2q33.3, 2q37.3, 3p12.3, 4q35.2, and 13q34 in SCC. Many of the loss regions in the chr3, -5, -9, -13, and -17 loss that occur in SCC patients carry known tumor suppressors, such as TP53, RB1, and APC [6–8]. The epidermal growth factor receptor (EGFR) is a transmembrane glycoprotein, located on 7p12, that conducts signals to downstream cascades such as PI3K-AKT and RAS-RAF-MEK-ERK. It has been observed that high-frequency copy number gains and the overexpression of proteins occur in SCC cases [9, 10]. An in-frame deletion of exons 2 to 7 in EGFR was found in three SCC cases and resulted in the development of NSCLC in a mouse model [11, 12]. The PI3K-AKT and RAS-RAF-MEK-ERK signaling pathways play central roles in antiapoptosis and proliferation in many cancers, including SCC [13–15]. Kirsten rat sarcoma viral oncogene homolog (KRAS), located on chr12p12.1, belongs to the canonical RAS family, which also includes HRA and NRAS. The three conserved RAS genes encode monomeric GTPases and have been found to be frequent mutations in approximately 30% of all cancers [16]. RAS receives stimulation from upstream receptors such as EGFR and conducts signals to the downstream pathways to regulate diverse cellular responses, including cell proliferation, differentiation, and apoptosis. Many proteins, including SOS, RAF, and MEK, participate in the process of signal transmission, and their dysfunction may leave the whole pathway dysregulated [17, 18]. Mutations of KRAS, NRAS, and HRAS were reported in lung cancer, including squamous cell carcinoma [19, 20]. The PI3K/AKT pathway also can be activated by RAS to promote cell escape from programs. The dysregulation of the RAS/ERK and PI3K/AKT pathways is common in many cancer types [18, 21]. Phosphatidylinositol-4,5-bisphosphate 3-kinase, catalytic subunit alpha (PIK3CA), encoding the catalytic subunit of PI3K, is located on the 3q26 amplified area and is found with high-frequency copy number (CN) gains and novel mutations in SCC [22–25]. Amplification of CN and somatic mutation presumably activate the PI3K pathway, leading to AKT activation, and provide tumor cells with multiple tumor-specific characteristics such as apoptosis arrest and limitless replicative potential [13, 26]. However, the detailed mechanisms are not clear. V-Akt murine thymoma viral oncogene homolog 1 (AKT1), located on 14q32, is one of three closely related serine-threonine kinases (AKT1, AKT2, and AKT3). The E17K mutation of AKT1, resulting in activation of the kinase, was found in 5.5% of SCC patients [27]. In SCC, aberrant amplification or expression of FGFR1 and IGF1R leads to the dysfunction of downstream signaling via the PI3K/AKT and RAS/MEK pathways [28–32]. Deletions involving chr3p and 9p21 in lung cancer, including SCC, were reported by The Cancer Genome Atlas (TCGA) [33, 34]. Some known tumor suppressor genes such as RASSF1A, FUS1, FHIT, and CDKN2A are located in these regions, and the loss of the allele may lead to aberrant tumorigenesis [35].

MicroRNAs (miRNAs) are a class of endogenous 20–25 nucleotide small RNAs that regulate mRNA translation and degradation through perfect or nearly perfect complementarity to the 3′ untranslated regions (UTRs) of the target mRNA [36–40]. In Cho's review [41], many miRNAs can be cancer biomarkers. The target genes regulated by miRNAs have been demonstrated to play critical roles in cancer pathogenesis [42–45]. For instance, Jiang et al. reported that non-small-cell lung cancer patients with higher BCL11A expression have better prognosis, and the expression of BCL11A is regulated by microRNA-30a. Approximately 1%–3% of the genome codes for microRNA sequences and 30% of known genes are presumably regulated by miRNA [46]. The miRNA let-7 was first found in* Caenorhabditis elegans,* and emerging evidence suggests its critical role in lung cancer [47]. In vitro and in vivo, reduced expression levels of let-7 have been observed in lung cancer, and the downregulated expression of let-7 was associated with shortened postoperative survival of lung cancer patients. In A549, a lung adenocarcinoma cell line, the proliferation of cancer cells was inhibited by the overexpression of let-7 [48]. The 3′UTR of RAS, a key oncogene in lung cancer, has complementary sites to let-7. In lung cancer, it has been shown that the level of let-7 is reduced and the expression of RAS protein is increased significantly, suggesting a mechanism in which RAS is the target of let-7 [49]. In addition to RAS, target complementary sites to let-7 in the 3′UTR of c-myc and high mobility group protein A2 (HMGA2) were found to inhibit tumorigenesis [50–53]. In different lung cancer subtypes, miR-205, miR-93, and miR-221 were uniquely increased in the SCC, but let-7e was downregulated both in lung adenocarcinoma (AD) and in SCC [54]. The epithelial to mesenchymal transition is suppressed by miRNA205 targeting of the transcriptional factors ZEB1 and SIP1 [55]. In addition, the tumor suppressor genes IL24 and IL32 may be targets of miR-205 [56]. Xing et al. identified three miRNAs (miR-205, miR-210, and miR-708) to discriminate SCC from healthy individuals (73% sensitivity and 96% specificity) [57]. In SCC, the expression of miR-146b and miR-155 is associated with the overall survival rate [58]. Plasma miR-21 was identified as an early detection and chemosensitivity biomarker in NSCLC [59]. In glioblastoma cell lines, miR-21, as an antiapoptotic factor, exhibits aberrant high expression, and knockdown of it resulted in the activation of cell apoptosis caspases [60]. Further, miRNAs including miR-182, miR-486-5p, miR-30a, miR-140-3p, miR-31, miR-34a, miR-25, and miR-191 play tumor-suppressor roles in lung cancer [57]. Above all, miRNAs can be useful prognostic predictors of SCC to help us to understand SCC pathogenesis.

In this study, by means of regression analysis, we speculated that various factors such as copy number variation (CNV), miRNA, and methylation could be related to the regulation of each gene. Then, we collect SCC related genes from the hsa05225 pathway (non-small-cell lung cancer,* Homo sapiens*) in KEGG and obtained the regulatory factors, as discussed above. Based on the Bayesian network, we rebuilt a novel key net between the SCC genes and their regulatory factors. In this net, we identified some novel genes and miRNAs involved in SCC pathogenesis and some known genes or factors that were previously neglected and should receive more attention.

## 2. Methods

### 2.1. Datasets

The gene expression, microRNA expression, and DNA methylation data of lung cancer patients were downloaded from TCGA (The Cancer Genome Atlas) squamous cell lung carcinoma project [61] website (https://tcga-data.nci.nih.gov/docs/publications/lusc_2012/). The expression level of 18,979 genes was measured with RNA-Seq and transformed into log_2_ scale. The expression level of 437 microRNAs was measured with microRNA-Seq and also transformed into log_2_ scale. The DNA methylation level of 18,498 genes was measured using infimum.

The GISTIC [62] processed copy number variation by gene data was downloaded from PANGeA [63] (http://cbio.mskcc.org/cancergenomics/pancan_tcga/) and included 24,174 genes.

Only the 129 squamous cell lung carcinoma patients with all four types of data, that is, gene expression, microRNA expression, DNA methylation, and copy number variation, were analyzed. Their TCGA sample IDs are provided in Table 1.

### 2.2. Construction of Whole Genome Integrative Network

For each gene, its expression level was considered as the dependent variable, and all other factors including microRNA expression levels, methylations, and copy number variations of all genes were considered as independent variables. The network construction was decomposed into 18,979 regression models.

For each regression model, there were 437 + 18498 + 24174 = 43109 feature variables. We applied the variance inflation factor (VIF) regression algorithm [64] to build this large-scale regression model. VIF regression can not only fit the model as accurately as sophisticated but slow methods such as LASSO but also perform fast feature selection. It evaluates the prediction potential of each feature variable and then performs forward feature selection, also known as incremental feature selection (IFS). IFS has been widely used to solve high dimensional regression [65] and classification problems [66–69]. The VIF regression program was downloaded from http://cran.r-project.org/web/packages/VIF/.

By combining the VIF regression models that passed the adjusted *R* ^2^ [65] cutoff 0.4, we obtained a whole genome integrative network in which the expression of target genes was regulated by microRNA, methylation, and copy number variation.

### 2.3. Refined Key Bayesian Subnetwork of Lung Cancer

Using VIF regression, we constructed the backbone network structure. However, the network was too complex for further functional analysis. Network decomposition technologies [70] were required to obtain the key subnetwork. Another problem was how to fully utilize the existing knowledge about lung cancer to discover novel key genes. A third was that the regression model did not consider the regulation structure between feature variables and therefore included false positive regulations. For example, we found that A and B regulate C, but the actual regulations may be that A regulates B, and then B regulates C.

To address these problems, first, we extracted the hsa05225 pathway (non-small-cell lung cancer,* Homo sapiens*) from KEGG using KEGGgraph [71]. Then, we used these known regulations as prior knowledge to perform Bayesian network analysis of the KEGG lung cancer genes and their candidate regulators identified by VIF regression. The Bayesian network of KEGG lung cancer genes and their candidate regulators expanded the key lung cancer subnetwork and provided novel key genes, which may enrich the understanding of lung cancer mechanisms. Furthermore, it refined the regulations among KEGG lung cancer genes and their candidate regulators and reduced the false discovery rate. We used the *R* package bnlearn [72] (http://www.bnlearn.com/) to build the Bayesian network.

## 3. Results and Discussion

### 3.1. The Novel Candidate Key Drivers of Lung Cancer

**Figure 1:**
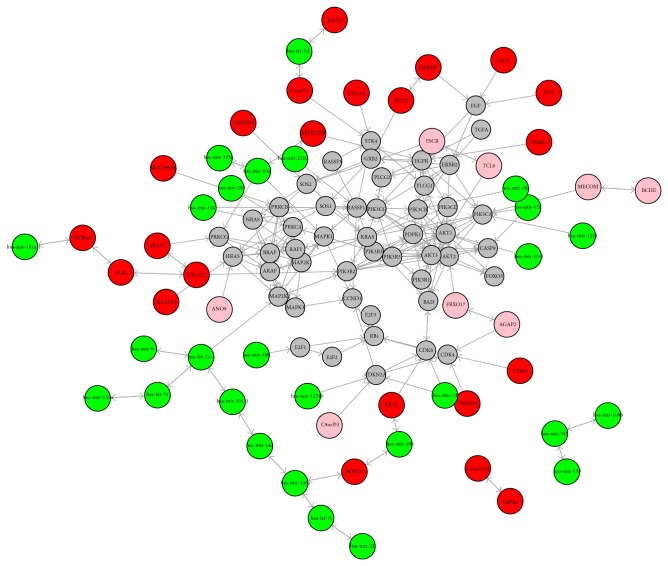
The refined key Bayesian subnetwork of lung cancer. The grey, green, red, and pink nodes represent mRNA genes, microRNAs, methylations, and copy number variations (CNVs), respectively. The one-arrow edges represent directed regulation, while the two-arrow edges represent undirected regulation.

We used VIF regression to build the whole genome integrative network of genes, microRNAs, methylations, and copy number variations. Then, the subnetwork involving the known lung cancers from KEGG was isolated and refined using the Bayesian network method. The refined key Bayesian subnetwork of lung cancer is shown in Figure 1. There were 48 mRNA genes, 27 microRNAs, 22 methylations, and 8 copy number variations. This integrative Bayesian network facilitates the investigation of the roles of microRNAs, methylations, and copy number variations in lung cancer. It reflects the complex lung cancer pathways that involve multiple levels of components. The key drivers of this network can serve as prognosis biomarkers and therapeutic drug targets.

From Figure 1, we identified some novel key drivers such as HOXD3, ARHGDIB, AGAP2, let-7a, and miR-31 that played important roles in the pathogenesis of lung cancer on the gene, microRNA, methylation, and copy number variation levels.

### 3.2. The Biological Roles of Novel Candidate Methylation Key Drivers

Based on our analysis, the methylation of HOXD3 interacts with the expressions of hsa-mir-100 and hsa-mir-146a and therefore indirectly affects many neighbor genes and microRNAs in the network.

Homeobox D3 (HOXD3) is a member of the highly conserved homeobox family, which possesses four similar clusters, HOXA, HOXB, HOXC, and HOXD. HOX genes have been reported to play important roles in cell adhesion, cell apoptosis and differentiation, and multiple receptor signaling [73, 74], and their aberrant expression occurs in many cancers originating from prostate, stomach, and lung [75–79]. Overexpression of HOXD3 enhanced motility and invasiveness in A549 cells [80]. In NSCLC, miR-100, targeted to polo-like kinase 1 (PLK1), was significantly reduced in expression and correlated with clinical stage [81]. In small-cell lung cancer (SCLC), it has been proved that HOXA1 is targeted and regulated by miR-100, and the expression of HOXA1 is inversely correlated with miR-100 [82]. Our bioinformatic analysis revealed that miR-100 and miR-146a are putative novel regulators to HOXD3 as well as HOXA1. In addition, it revealed that keratin 8 (KRT8), which participates in forming intermediate-sized filaments, is another target of miR-100 and is involved in the cell cycle controlled by CDK6. In NSCLC, miR-146a acts as a tumor suppressor by inhibiting cell growth and migration and inducing apoptosis by targeting EGFR, which plays a critical part in SCC pathogenesis [83]. It was demonstrated that miR-146a regulates multiple targets to affect different aspects of the central network.

As shown in Figure 1, another key methylation driver was ARHGDIB, which interacts with the mRNA expression of EGFR and is associated with the expression of hsa-mir-10a.

It is known that Rho GDP dissociation inhibitor (GDI) beta (RhoGDI, also known as ARHGDIB) is a guanine nucleotide dissociation inhibitor specific to the Rho family of small GTPases. RhoGDI2 is involved in diverse cellular events, such as cell signaling, cell proliferation, and cytoskeletal organization. In different cancer types, RhoGDI2 performs diverse functions and exhibits different aberrant expression levels. In ovarian and stomach cancer, the expression of RhoGDI2 is upregulated, while it is downregulated in bladder cancer and lung cancer [84–88]. It has been observed that RhoGDI2 expression is downregulated in lung cancer, and the lower expression is strongly correlated with higher malignancy grade, lower cell differentiation, and greater lymph node metastasis of lung cancer. The expression of RhoGDI2 is inversely correlated to the activation level of the PI3K/Akt/mTOR pathway, which plays key roles in SCC [88]. In bladder cancer, c-Src protein phosphorylates RhoGDI2 and is regulated by EGFR [89–91]. We also found a novel correlation between RhoGDI2 and EGFR. RhoGDI2 is involved in the EGFR-associated key network in SCC directly or indirectly through interaction with c-Src. In addition, the results of our analysis reveal that RhoGDI2 is regulated by miR-10a. There is no evidence that RhoGDI2 is the direct target of miR-10a, and more focus is needed on this subnet of miR-10a-RhoGDI2-EGFR. As discussed above, RhoGDI2 may be a putative molecular marker in metastatic lung cancer, which warrants deeper investigation for validation.

**Figure 2:**
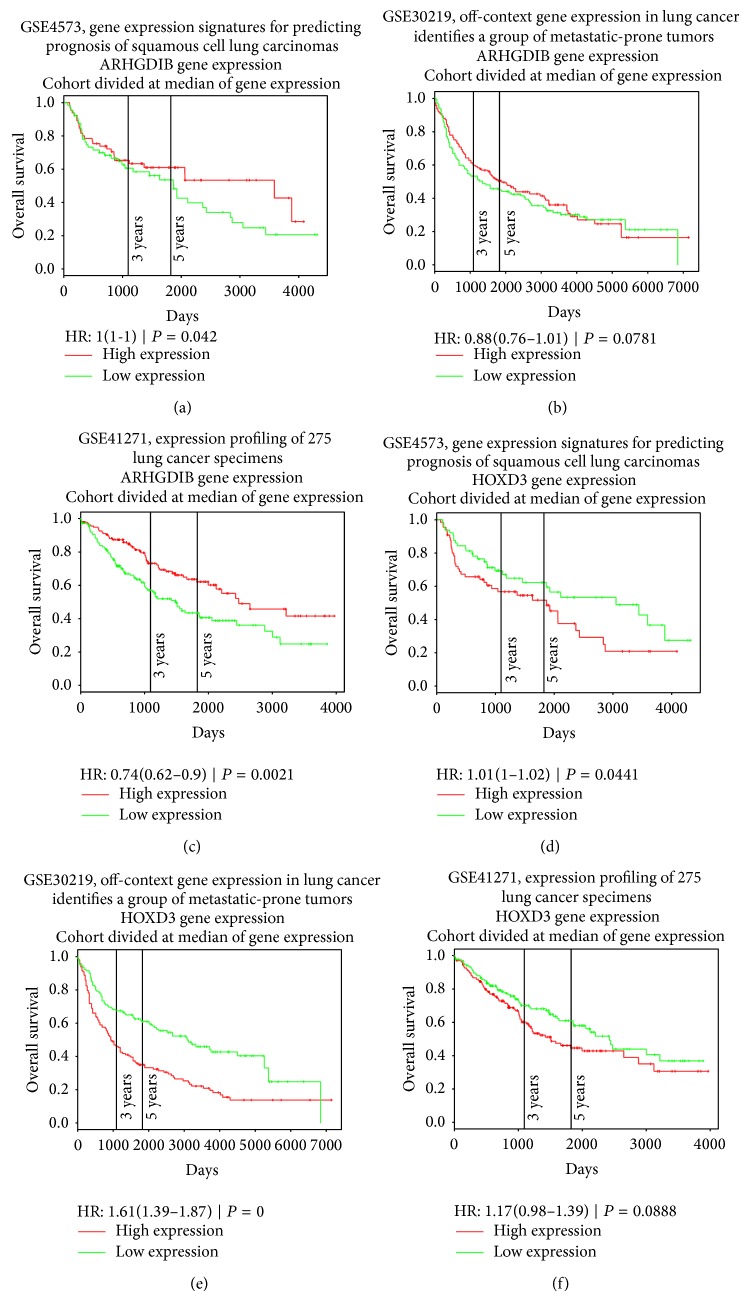
The Kaplan-Meier plots of key drivers ARHGDIB and HOXD3 on three large lung cancer datasets. The log-rank *P* values of ARHGDIB on GSE4573 (a), GSE30219 (b), and GSE41271 (c) were 0.042, 0.0781, and 0.0021, respectively. The patients with high expression of ARHGDIB had good prognoses. The log-rank *P* values of HOXD3 on GSE4573 (d), GSE30219 (e), and GSE41271 (f) were 0.0441, 0, and 0.0888, respectively. The patients with high expression of HOXD3 had poor prognoses.

It is known that DNA methylation suppresses gene expression [92]. To investigate whether the gene expression levels of these methylation key drivers, ARHGDIB and HOXD3, play significant roles in prognosis, we collected three large lung cancer survival datasets from PROGgene [93]: GSE4573, which included 129 squamous cell lung carcinomas patients, GSE30219, with 281 lung cancer samples, and GSE41271, with 275 lung cancer specimens. The patients were divided into high expression group and low expression group by the median. The overall survival rates of the two groups were compared using the log-rank test [94], and their Kaplan-Meier plots [95] are shown in Figure 2. The log-rank *P* values of ARHGDIB on GSE4573, GSE30219, and GSE41271 were 0.042, 0.0781, and 0.0021, respectively. The patients with high expression of ARHGDIB had good prognoses. Meanwhile, the log-rank *P* values of HOXD3 on GSE4573, GSE30219, and GSE41271 were 0.0441, 0, and 0.0888, respectively. The patients with high expression of HOXD3 had poor prognoses. The key drivers, ARHGDIB and HOXD3, play significant roles in prognosis prediction.

### 3.3. The Biological Roles of Novel Candidate MicroRNA Key Drivers

In addition to novel SCC related mRNA/methylation/CNV genes, we also observed some novel miRNAs to be involved in the central network of SCC pathogenesis, such as let-7a and miR-31.

In a study of Chinese SCC patients, miR-31 was considered as a new prognostic biomarker for SCC [96]. The miR-31, located on chr9p21.3 in genome, is involved in cell proliferation, migration, and invasion. In breast cancer, miR-31 was considered as a suppressor of metastasis [97]. In mesothelioma (MM) cells, the reintroduction of miR-31 reveals activity that suppresses the cell cycle and migration [98]. In our new key network, mir-31 is closely associated with CDKN2A and CDK. It has been reported that the chr9q21.3 region is frequently and homozygously deleted in MM, and the deletion is linked to a known tumor suppressor gene, CDKN2A [99–102]. In approximately 31% of NSCLC, the deletion of chr9p21.3 was observed [103]. All this evidence indicates that the codeletion of miR-31 with CDKN2A occurs normally in cancers, and both are beneficial to tumor pathogenesis and metastasis. CDKN2A can bond to CDK6 to suppress the cell cycle process directly. We speculate that miR-31 regulates the CDK6-related cell cycle directly or indirectly in SCC and is an excellent diagnostic biomarker for SCC.

It has been reported that the let-7 microRNA family plays critical roles in pathogenesis of SCC. We also found new miRNAs of the let-7 family to be highlighted in our new network, such as let-7a, let-7e, and let-7i, which indicated that more let-7 family members than we previously knew function in SCC tumorigenesis. Let-7a was characterized as a tumor suppressor in various cancers, including lung and colon cancer [104–108]. It has been observed that the expression of let-7 is downregulated in many cancer types and is correlated with poor clinical prognosis [48, 106]. In NSCLC cell lines, let-7a inhibits cell proliferation and invasion by interacting with K-Ras and HMGA2 [104]. Target analysis reveals that IGF1R is a target gene, one of let-7's numerous targets, and is involved in the IGF1R/RAS/MAPK/ELK1 pathway, playing major roles in cell proliferation and cell survival [109]. In ovarian cancer, it was reported that let-7e has many target genes including HMGA2, C14ofr28, LIN28B, and ARID3B, and let-7e expression is lower than in adjacent tissues [110]. It has also been reported that let-7i is significantly downregulated in SCC [111]. In our network, we found that let-7 family members appeared frequently. Some let-7 members interact with their putative targets genes directly, and some members function in the central net indirectly through the regulation of other miRNA transcripts, including mir-101-1 and mir-146a. All this evidence implies that the let-7-miRNA subnet is worth greater attention and may be a novel marker for SCC.

### 3.4. The Biological Roles of Novel Candidate CNV Key Drivers

From Figure 1, we can see that the CNV of AGAP2 can regulate the gene expression of CDK4 and is associated with the CNV of FBXO17.

As we know, AGAP2 (ArfGAP With GTPase Domain, Ankyrin Repeat And PH Domain 2), also known as PIKE, belongs to the centaurin GRPase family, including three members, PIKE-L, PIKE-S, and PIKE-A [112–114]. PIKE-S and PIKE-L enhance PI3K antiapoptosis activity, but PIKE-A promotes the downstream Akt instead [113, 115]. PIKE proteins participate in regulating the signal transducer and activator of transcription 5A (STAT5) by Janus kinase 2 (JAK2) phosphorylation [116]. Because of gene amplification, PIKE-A is overexpressed in glioblastoma and astrocytoma [113]. Its increased expression has been observed in various cancer types including breast, prostate, skin, colon, ovary, liver, stomach, lung, and kidney [117–120]. In the genome, the PIKE gene is adjacent to CDK4, which plays a key role in cell cycle control [121, 122]. As a component of the CDK4 amplicon, coamplification of CDK4 and CENTG1 has been frequently found in various cancers [123–127]. An integrated oncomir/oncogene DNA cluster, comprised of PIKE-A, CDK4, and has-miR26a, accelerates the aggressiveness in glioblastoma [128]. CDK4 and PIKEs are involved concomitantly in cancer pathogenesis by cooperatively targeting the PI3K/AKT and Rb1 pathway, although some studies have shown that overexpression of PIKEs alone can elicit NIH3T3 cell transformation [117, 128]. In our study, a relationship was found between CDK4 and PIKEs. This result demonstrated that their combination is more indirectly responsive to PI3K pathway dysfunction in SCC. The direct interaction between CDK4 and PIKEs requires deeper experiments to validate. The supposed effects between them may exist at different levels, including genome, transcriptome, and proteome, and more experimental evidence is needed to support these effects.

## 4. Conclusion

In our study, we established a new key network for SCC to help us mine the mechanisms more effectively. We not only found novel SCC related genes and subnets but also noticed significant changes to the old net. Compared to past studies, EGR-EGFR, PIK3CG/PIK3CA, HRAS, CDK6, CDK4, and CDKN2A were found to have interactions and relationships with more genes and miRNAs, expanding the network's scope and depth. These proteins may be the hinge of the whole pathogenesis network and warrant greater focus.

## Figures and Tables

**Table 1:** TCGA sample IDs of 129 squamous cell lung carcinoma patients with gene expression, microRNA expression, DNA methylation, and CNV data.

Sample IDs	Sample IDs	Sample IDs	Sample IDs	Sample IDs
TCGA-18-3414	TCGA-33-4566	TCGA-66-2795	TCGA-39-5021	TCGA-39-5037
TCGA-18-3411	TCGA-60-2713	TCGA-66-2744	TCGA-51-4080	TCGA-37-4141
TCGA-18-3410	TCGA-18-3417	TCGA-66-2742	TCGA-21-1075	TCGA-33-4547
TCGA-18-3412	TCGA-66-2786	TCGA-66-2771	TCGA-39-5031	TCGA-33-4582
TCGA-22-0944	TCGA-33-4583	TCGA-66-2787	TCGA-21-1077	TCGA-34-5240
TCGA-46-3766	TCGA-66-2785	TCGA-46-3767	TCGA-37-4135	TCGA-66-2791
TCGA-18-3406	TCGA-66-2780	TCGA-66-2754	TCGA-21-1072	TCGA-66-2789
TCGA-22-4604	TCGA-66-2781	TCGA-18-3415	TCGA-37-4133	TCGA-66-2788
TCGA-60-2706	TCGA-18-4721	TCGA-18-3419	TCGA-33-4533	TCGA-66-2792
TCGA-60-2696	TCGA-22-4613	TCGA-46-3765	TCGA-33-4538	TCGA-22-4607
TCGA-60-2710	TCGA-22-4601	TCGA-18-3421	TCGA-37-4130	TCGA-22-4596
TCGA-60-2698	TCGA-66-2782	TCGA-18-3408	TCGA-66-2790	TCGA-33-4532
TCGA-60-2711	TCGA-43-3920	TCGA-18-3416	TCGA-66-2737	TCGA-22-4595
TCGA-60-2712	TCGA-46-3769	TCGA-66-2793	TCGA-66-2753	TCGA-22-4591
TCGA-60-2708	TCGA-43-3394	TCGA-66-2783	TCGA-66-2734	TCGA-22-4594
TCGA-66-2758	TCGA-51-4079	TCGA-66-2794	TCGA-34-2596	TCGA-37-3789
TCGA-60-2722	TCGA-37-3792	TCGA-66-2800	TCGA-34-2608	TCGA-46-3768
TCGA-60-2721	TCGA-39-5039	TCGA-56-1622	TCGA-43-2581	TCGA-43-2578
TCGA-60-2724	TCGA-63-5131	TCGA-60-2725	TCGA-60-2695	TCGA-66-2727
TCGA-60-2716	TCGA-39-5036	TCGA-51-4081	TCGA-34-2600	TCGA-39-5011
TCGA-66-2759	TCGA-39-5034	TCGA-22-1012	TCGA-66-2766	TCGA-34-5241
TCGA-60-2723	TCGA-63-5128	TCGA-22-1011	TCGA-66-2767	TCGA-39-5029
TCGA-66-2755	TCGA-39-5035	TCGA-21-1079	TCGA-66-2770	TCGA-39-5028
TCGA-60-2719	TCGA-39-5030	TCGA-21-1078	TCGA-66-2765	TCGA-18-3407
TCGA-60-2720	TCGA-66-2768	TCGA-21-1080	TCGA-66-2763	TCGA-18-4086
TCGA-60-2714	TCGA-33-4586	TCGA-21-1076	TCGA-66-2777	

## References

[1] Ramalingam S., Pawlish K., Gadgeel S., Demers R., Kalemkerian G. P. (1998). Lung cancer in young patients: analysis of a surveillance, epidemiology, and end results database. *Journal of Clinical Oncology*.

[2] Miller Y. E. (2005). Pathogenesis of lung cancer: 100 Year report. *American Journal of Respiratory Cell and Molecular Biology*.

[3] Travis W. D. (2011). Pathology of lung cancer. *Clinics in Chest Medicine*.

[4] Sriram K. B., Larsen J. E., Savarimuthu Francis S. M. (2012). Array-comparative genomic hybridization reveals loss of SOCS6 is associated with poor prognosis in primary lung squamous cell carcinoma. *PLoS ONE*.

[5] Son J. W., Jeong K. J., Jean W.-S. (2011). Genome-wide combination profiling of DNA copy number and methylation for deciphering biomarkers in non-small cell lung cancer patients. *Cancer Letters*.

[6] Wistuba I. I., Behrens C., Milchgrub S. (1999). Sequential molecular abnormalities are involved in the multistage development of squamous cell lung carcinoma. *Oncogene*.

[7] Wistuba I. I., Behrens C., Virmani A. K. (2000). High resolution chromosome 3p allelotyping of human lung cancer and preneoplastic/preinvasive bronchial epithelium reveals multiple, discontinuous sites of 3p allele loss and three regions of frequent breakpoints. *Cancer Research*.

[8] Drilon A., Rekhtman N., Ladanyi M., Paik P. (2012). Squamous-cell carcinomas of the lung: emerging biology, controversies, and the promise of targeted therapy. *The Lancet Oncology*.

[9] Hirsch F. R., Varella-Garcia M., Bunn P. A. (2003). Epidermal growth factor receptor in non-small-cell lung carcinomas: correlation between gene copy number and protein expression and impact on prognosis. *Journal of Clinical Oncology*.

[10] Dacic S., Flanagan M., Cieply K. (2006). Significance of EGFR protein expression and gene amplification in non-small cell lung carcinoma. *American Journal of Clinical Pathology*.

[11] Ekstrand A. J., Sugawa N., James C. D., Collins V. P. (1992). Amplified and rearranged epidermal growth factor receptor genes in human glioblastomas reveal deletions of sequences encoding portions of the N- and/or C-terminal tails. *Proceedings of the National Academy of Sciences of the United States of America*.

[12] Ji H., Zhao X., Yuza Y. (2006). Epidermal growth factor receptor variant III mutations in lung tumorigenesis and sensitivity to tyrosine kinase inhibitors. *Proceedings of the National Academy of Sciences of the United States of America*.

[13] Heist R. S., Sequist L. V., Engelman J. A. (2012). Genetic changes in squamous cell lung cancer: a review. *Journal of Thoracic Oncology*.

[14] Karnoub A. E., Weinberg R. A. (2008). Ras oncogenes: split personalities. *Nature Reviews Molecular Cell Biology*.

[15] Sowa Y., Sakai T. (2012). Ras/Raf/MEK/ERK cascade. *Nihon Rinsho. Japanese Journal of Clinical Medicine*.

[16] Bos J. L. (1989). Ras oncogenes in human cancer: a review. *Cancer Research*.

[17] Westcott P. M. K., To M. D. (2013). The genetics and biology of KRAS in lung cancer. *Chinese Journal of Cancer*.

[18] Seger R., Krebs E. G. (1995). The MAPK signaling cascade. *The FASEB Journal*.

[19] Suzuki Y., Orita M., Shiraishi M., Hayashi K., Sekiya T. (1990). Detection of ras gene mutations in human lung cancers by single-strand conformation polymorphism analysis of polymerase chain reaction products. *Oncogene*.

[20] Mitsudomi T., Viallet J., Mulshine J. L., Linnoila R. I., Minna J. D., Gazdar A. F. (1991). Mutations of ras genes distinguish a subset of non-small-cell lung cancer cell lines from small-cell lung cancer cell lines. *Oncogene*.

[21] Engelman J. A., Luo J., Cantley L. C. (2006). The evolution of phosphatidylinositol 3-kinases as regulators of growth and metabolism. *Nature Reviews Genetics*.

[22] Yamamoto H., Shigematsu H., Nomura M. (2008). PIK3CA mutations and copy number gains in human lung cancers. *Cancer Research*.

[23] Kawano O., Sasaki H., Okuda K. (2007). PIK3CA gene amplification in Japanese non-small cell lung cancer. *Lung Cancer*.

[24] Samuels Y., Wang Z., Bardelli A. (2004). High frequency of mutations of the PIK3CA gene in human cancers. *Science*.

[25] Kawano O., Sasaki H., Endo K. (2006). PIK3CA mutation status in Japanese lung cancer patients. *Lung Cancer*.

[26] Vivanco I., Sawyers C. L. (2002). The phosphatidylinositol 3-kinase-AKT pathway in human cancer. *Nature Reviews Cancer*.

[27] Malanga D., Scrima M., de Marco C. (2008). Activating E17K mutation in the gene encoding the protein kinase AKT1 in a subset of squamous cell carcinoma of the lung. *Cell Cycle*.

[28] Weiss J., Sos M. L., Seidel D. (2010). Frequent and focal FGFR1 amplification associates with therapeutically tractable FGFR1 dependency in squamous cell lung cancer. *Science Translational Medicine*.

[29] Mason I. (2007). Initiation to end point: the multiple roles of fibroblast growth factors in neural development. *Nature Reviews Neuroscience*.

[30] Dziadziuszko R., Merrick D. T., Witta S. E. (2010). Insulin-like growth factor receptor 1 (IGF1R) gene copy number is associated with survival in operable non-small-cell lung cancer: a comparison between IGF1R fluorescent in situ hybridization, protein expression, and mRNA expression. *Journal of Clinical Oncology*.

[31] Cappuzzo F., Tallini G., Finocchiaro G. (2009). Insulin-like growth factor receptor 1 (IGF1R) expression and survival in surgically resected non-small-cell lung cancer (NSCLC) patients. *Annals of Oncology*.

[32] Gualberto A., Dolled-Filhart M., Gustavson M. (2010). Molecular analysis of non-small cell lung cancer identifies subsets with different sensitivity to insulin-like growth factor I receptor inhibition. *Clinical Cancer Research*.

[33] Cancer Genome Atlas Research Network (2012). Comprehensive genomic characterization of squamous cell lung cancers. *Nature*.

[34] Zabarovsky E. R., Lerman M. I., Minna J. D. (2002). Tumor suppressor genes on chromosome 3p involved in the pathogenesis of lung and other cancers. *Oncogene*.

[35] Rooney M., Devarakonda S., Govindana R. (2013). Genomics of squamous cell lung cancer. *Oncologist*.

[36] de Moor C. H., Meijer H., Lissenden S. (2005). Mechanisms of translational control by the 3′ UTR in development and differentiation. *Seminars in Cell and Developmental Biology*.

[37] Lai E. C. (2002). Micro RNAs are complementary to 3′ UTR sequence motifs that mediate negative post-transcriptional regulation. *Nature Genetics*.

[38] Robins H., Press W. H. (2005). Human microRNAs target a functionally distinct population of genes with AT-rich 3′ UTRs. *Proceedings of the National Academy of Sciences of the United States of America*.

[39] Stark A., Brennecke J., Bushati N., Russell R. B., Cohen S. M. (2005). Animal microRNAs confer robustness to gene expression and have a significant impact on 3′UTR evolution. *Cell*.

[40] Sun M., Hurst L. D., Carmichael G. G., Chen J. (2005). Evidence for a preferential targeting of 3′-UTRs by cis-encoded natural antisense transcripts. *Nucleic Acids Research*.

[41] Cho W. C. S. (2010). MicroRNAs: potential biomarkers for cancer diagnosis, prognosis and targets for therapy. *International Journal of Biochemistry and Cell Biology*.

[42] He L., He X., Lowe S. W., Hannon G. J. (2007). MicroRNAs join the p53 network—another piece in the tumour-suppression puzzle. *Nature Reviews Cancer*.

[43] Cho W. C. S. (2007). OncomiRs: the discovery and progress of microRNAs in cancers. *Molecular Cancer*.

[44] Hwang H.-W., Mendell J. T. (2006). MicroRNAs in cell proliferation, cell death, and tumorigenesis. *British Journal of Cancer*.

[45] Esquela-Kerscher A., Slack F. J. (2006). Oncomirs—microRNAs with a role in cancer. *Nature Reviews Cancer*.

[46] Lai E. C., Tomancak P., Williams R. W., Rubin G. M. (2003). Computational identification of Drosophila microRNA genes. *Genome Biology*.

[47] Reinhart B. J., Slack F. J., Basson M. (2000). The 21-nucleotide let-7 RNA regulates developmental timing in *Caenorhabditis elegans*. *Nature*.

[48] Takamizawa J., Konishi H., Yanagisawa K. (2004). Reduced expression of the let-7 microRNAs in human lung cancers in association with shortened postoperative survival. *Cancer Research*.

[49] Johnson S. M., Grosshans H., Shingara J. (2005). RAS is regulated by the let-7 microRNA family. *Cell*.

[50] Mayr C., Hemann M. T., Bartel D. P. (2007). Disrupting the pairing between let-7 and Hmga2 enhances oncogenic transformation. *Science*.

[51] Lee Y. S., Dutta A. (2007). The tumor suppressor microRNA let-7 represses the HMGA2 oncogene. *Genes and Development*.

[52] He X. Y., Chen J. X., Zhang Z., Li C. L., Peng Q. L., Peng H. M. (2010). The let-7a microRNA protects from growth of lung carcinoma by suppression of k-Ras and c-Myc in nude mice. *Journal of Cancer Research and Clinical Oncology*.

[53] Hyeon H. K., Kuwano Y., Srikantan S., Eun K. L., Martindale J. L., Gorospe M. (2009). HuR recruits let-7/RISC to repress c-Myc expression. *Genes and Development*.

[54] Zhang Y.-K., Zhu W.-Y., He J.-Y. (2012). MiRNAs expression profiling to distinguish lung squamous-cell carcinoma from adenocarcinoma subtypes. *Journal of Cancer Research and Clinical Oncology*.

[55] Gregory P. A., Bert A. G., Paterson E. L. (2008). The miR-200 family and miR-205 regulate epithelial to mesenchymal transition by targeting ZEB1 and SIP1. *Nature Cell Biology*.

[56] Majid S., Dar A. A., Saini S. (2010). MicroRNA-205-directed transcriptional activation of tumor suppressor genes in prostate cancer. *Cancer*.

[57] Xing L., Todd N. W., Yu L., Fang H., Jiang F. (2010). Early detection of squamous cell lung cancer in sputum by a panel of microRNA markers. *Modern Pathology*.

[58] Yanaihara N., Caplen N., Bowman E. (2006). Unique microRNA molecular profiles in lung cancer diagnosis and prognosis. *Cancer Cell*.

[59] Wei J., Gao W., Zhu C.-J. (2011). Identification of plasma microRNA-21 as a biomarker for early detection and chemosensitivity of non-small cell lung cancer. *Chinese Journal of Cancer*.

[60] Chan J. A., Krichevsky A. M., Kosik K. S. (2005). MicroRNA-21 is an antiapoptotic factor in human glioblastoma cells. *Cancer Research*.

[61] Cancer Genome Atlas Research Network (2012). Comprehensive genomic characterization of squamous cell lung cancers. *Nature*.

[62] Mermel C. H., Schumacher S. E., Hill B., Meyerson M. L., Beroukhim R., Getz G. (2011). GISTIC2.0 facilitates sensitive and confident localization of the targets of focal somatic copy-number alteration in human cancers. *Genome Biology*.

[63] Ciriello G., Miller M. L., Aksoy B. A., Senbabaoglu Y., Schultz N., Sander C. (2013). Emerging landscape of oncogenic signatures across human cancers. *Nature Genetics*.

[64] Lin D., Foster D. P., Ungar L. H. (2011). VIF regression: a fast regression algorithm for large data. *Journal of the American Statistical Association*.

[65] Huang T., Liu L., Qian Z., Tu K., Li Y., Xie L. (2010). Using GeneReg to construct time delay gene regulatory networks. *BMC Research Notes*.

[66] Huang T., Wang C., Zhang G., Xie L., Li Y. (2012). SySAP: a system-level predictor of deleterious single amino acid polymorphisms. *Protein and Cell*.

[67] Huang T., Cai Y.-D. (2013). An information-theoretic machine learning approach to expression QTL analysis. *PLoS ONE*.

[68] Huang T., He Z.-S., Cui W.-R. (2013). A sequence-based approach for predicting protein disordered regions. *Protein and Peptide Letters*.

[69] Jiang Y., Huang T., Chen L., Gao Y. F., Cai Y., Chou K.-C. (2013). Signal propagation in protein interaction network during colorectal cancer progression. *BioMed Research International*.

[70] Huang T., Cui W., He Z.-S. (2009). Functional association between influenza A (H1N1) virus and human. *Biochemical and Biophysical Research Communications*.

[71] Zhang J. D., Wiemann S. (2009). KEGGgraph: a graph approach to KEGG PATHWAY in R and bioconductor. *Bioinformatics*.

[72] Scutari M. (2010). Learning Bayesian networks with the bnlearn R Package. *Journal of Statistical Software*.

[73] Cillo C., Cantile M., Faiella A., Boncinelli E. (2001). Homeobox genes in normal and malignant cells. *Journal of Cellular Physiology*.

[74] Shah N., Sukumar S. (2010). The Hox genes and their roles in oncogenesis. *Nature Reviews Cancer*.

[75] Graham A., Papalopulu N., Krumlauf R. (1989). The murine and *Drosophila homeobox* gene complexes have common features of organization and expression. *Cell*.

[76] Bach C., Buhl S., Mueller D., García-Cuéllar M.-P., Maethner E., Slany R. K. (2010). Leukemogenic transformation by HOXA cluster genes. *Blood*.

[77] Waltregny D., Alami Y., Clausse N., de Leval J., Castronovo V. (2002). Overexpression of the homeobox gene HOXC8 in human prostate cancer correlates with loss of tumor differentiation. *Prostate*.

[78] Calvo R., West J., Franklin W. (2000). Altered HOX and WNT7A expression in lung cancer. *Proceedings of the National Academy of Sciences of the United States of America*.

[79] Abe M., Hamada J.-I., Takahashi O. (2006). Disordered expression of HOX genes in human non-small cell lung cancer. *Oncology Reports*.

[80] Miyazaki Y. J., Hamada J.-I., Tada M. (2002). HOXD3 enhances motility and invasiveness through the TGF-*β*-dependent and -independent pathways in A549 cells. *Oncogene*.

[81] Liu J., Lu K.-H., Liu Z.-L., Sun M., De W., Wang Z.-X. (2012). MicroRNA-100 is a potential molecular marker of non-small cell lung cancer and functions as a tumor suppressor by targeting polo-like kinase 1. *BMC Cancer*.

[82] Xiao F., Bai Y., Chen Z. (2014). Downregulation of HOXA1 gene affects small cell lung cancer cell survival and chemoresistance under the regulation of miR-100. *European Journal of Cancer*.

[83] Chen G., Umelo I. A., Lv S. (2013). miR-146a inhibits cell growth, cell migration and induces apoptosis in non-small cell lung cancer cells. *PLoS ONE*.

[84] Stevens E. V., Banet N., Onesto C. (2011). RhoGDI2 antagonizes ovarian carcinoma growth, invasion and metastasis. *Small GTPases*.

[85] Zhen H., Yang S., Wu H. (2010). LyGDI is a promising biomarker for ovarian cancer. *International Journal of Gynecological Cancer*.

[86] Cho H. J., Baek K. E., Park S.-M. (2009). RhoGDI2 expression is associated with tumor growth and malignant progression of gastric cancer. *Clinical Cancer Research*.

[87] Gildea J. J., Seraj M. J., Oxford G. (2002). RhoGDI2 is an invasion and metastasis suppressor gene in human cancer. *Cancer Research*.

[88] Niu H., Li H., Xu C., He P. (2010). Expression profile of RhoGDI2 in lung cancers and role of RhoGDI2 in lung cancer metastasis. *Oncology Reports*.

[89] Thomas S. M., Brugge J. S. (1997). Cellular functions regulated by SRC family kinases. *Annual Review of Cell and Developmental Biology*.

[90] Said N., Theodorescu D. (2009). Pathways of metastasis suppression in bladder cancer. *Cancer and Metastasis Reviews*.

[91] Yamamoto N., Mammadova G., Song R. X. D., Fukami Y., Sato K.-I. (2006). Tyrosine phosphorylation of p145met mediated by EGFR and Src is required for serum-independent survival of human bladder carcinoma cells. *Journal of Cell Science*.

[92] Curradi M., Izzo A., Badaracco G., Landsberger N. (2002). Molecular mechanisms of gene silencing mediated by DNA methylation. *Molecular and Cellular Biology*.

[93] Goswami C. P., Nakshatri H. (2013). PROGgene: gene expression based survival analysis web application for multiple cancers. *Journal of Clinical Bioinformatics*.

[94] Harrington D. P., Fleming T. R. (1982). A class of rank test procedures for censored survival data. *Biometrika*.

[95] Andersen P. K., Gill R. D. (1982). Cox's regression model for counting processes: a large sample study. *The Annals of Statistics*.

[96] Tan X., Qin W., Zhang L. (2011). A 5-microRNA signature for lung squamous cell carcinoma diagnosis and hsa-miR-31 for prognosis. *Clinical Cancer Research*.

[97] Valastyan S., Reinhardt F., Benaich N. (2009). A pleiotropically acting microRNA, miR-31, inhibits breast cancer metastasis. *Cell*.

[98] Ivanov S. V., Goparaju C. M. V., Lopez P. (2010). Pro-tumorigenic effects of miR-31 loss in mesothelioma. *Journal of Biological Chemistry*.

[99] Ruas M., Peters G. (1998). The p16(INK4a)/CDKN2A tumor suppressor and its relatives. *Biochimica et Biophysica Acta*.

[100] Sharpless N. E. (2005). INK4a/ARF: a multifunctional tumor suppressor locus. *Mutation Research/Fundamental and Molecular Mechanisms of Mutagenesis*.

[101] Dacic S., Kothmaier H., Land S. (2008). Prognostic significance of p16/cdkn2a loss in pleural malignant mesotheliomas. *Virchows Archiv*.

[102] Krimpenfort P., IJpenberg A., Song J.-Y. (2007). p15Ink4b is a critical tumour suppressor in the absence of p16Ink4a. *Nature*.

[103] Nobori T., Szinai I., Amox D. (1993). Methylthioadenosine phosphorylase deficiency in human non-small cell lung cancers. *Cancer Research*.

[104] Wang Y. Y., Ren T., Cai Y. Y., He X. Y. (2013). MicroRNA let-7a inhibits the proliferation and invasion of nonsmall cell lung cancer cell line 95D by regulating K-RAS and HMGA2 gene expression. *Cancer Biotherapy and Radiopharmaceuticals*.

[105] Cai K., Wan Y., Sun G., Shi L., Bao X., Wang Z. (2012). Let-7a inhibits proliferation and induces apoptosis by targeting EZH2 in nasopharyngeal carcinoma cells. *Oncology Reports*.

[106] Dong Q., Meng P., Wang T. (2010). MicroRNA let-7a inhibits proliferation of human prostate cancer cells *In Vitro* and *In Viv*o by targeting E2F2 and CCND2. *PLoS ONE*.

[107] Esquela-Kerscher A., Trang P., Wiggins J. F. (2008). The let-7 microRNA reduces tumor growth in mouse models of lung cancer. *Cell Cycle*.

[108] Yang N., Kaur S., Volinia S. (2008). MicroRNA microarray identifies Let-7i as a novel biomarker and therapeutic target in human epithelial ovarian cancer. *Cancer Research*.

[109] Wang L.-N., Chen W.-W., Zhang J. (2013). The miRNA let-7a1 inhibits the expression of insulin-like growth factor 1 receptor (IGF1R) in prostate cancer PC-3 cells. *Asian Journal of Andrology*.

[110] Dahiya N., Sherman-Baust C. A., Wang T.-L. (2008). MicroRNA expression and identification of putative miRNA targets in ovarian cancer. *PLoS ONE*.

[111] Yang Y., Li X., Yang Q. (2010). The role of microRNA in human lung squamous cell carcinoma. *Cancer Genetics and Cytogenetics*.

[112] Jackson T. R., Kearns B. G., Theibert A. B. (2000). Cytohesins and centaurins: mediators of PI 3-kinase-regulated Arf signaling. *Trends in Biochemical Sciences*.

[113] Ahn J. Y., Rong R., Kroll T. G., van Meir E. G., Snyder S. H., Ye K. (2004). PIKE (phosphatidylinositol 3-kinase enhancer)-A GTPase stimulates Akt activity and mediates cellular invasion. *The Journal of Biological Chemistry*.

[114] Rong R., Ahn J.-Y., Huang H. (2003). PI3 kinase enhancer-Homer complex couples mGluRI to PI3 kinase, preventing neuronal apoptosis. *Nature Neuroscience*.

[115] Qi Q., Ye K. (2013). The roles of PIKE in tumorigenesis. *Acta Pharmacologica Sinica*.

[116] Chan C.-B., Liu X., Ensslin M. A. (2010). PIKE-A is required for prolactin-mediated STAT5a activation in mammary gland development. *The EMBO Journal*.

[117] Liu X., Hu Y., Hao C., Rempel S. A., Ye K. (2007). PIKE-A is a proto-oncogene promoting cell growth, transformation and invasion. *Oncogene*.

[118] Cai Y., Wang J., Li R., Ayala G., Ittmann M., Liu M. (2009). GGAP2/PIKE-A directly activates both the Akt and nuclear factor-*κ*B pathways and promotes prostate cancer progression. *Cancer Research*.

[119] Knobbe C. B., Trampe-Kieslich A., Reifenberger G. (2005). Genetic alteration and expression of the phosphoinositol-3-kinase/Akt pathway genes PIK3CA and PIKE in human glioblastomas. *Neuropathology and Applied Neurobiology*.

[120] Ahn J.-Y., Hu Y., Kroll T. G., Allard P., Ye K. (2004). PIKE-A is amplified in human cancers and prevents apoptosis by up-regulating Akt. *Proceedings of the National Academy of Sciences of the United States of America*.

[121] Collins V. P. (1995). Gene amplification in human gliomas. *Glia*.

[122] Reifenberger G., Reifenberger J., Ichimura K., Collins V. P. (1995). Amplification at 12q13-14 in human malignant gliomas is frequently accompanied by loss of heterozygosity at loci proximal and distal to the amplification site. *Cancer Research*.

[123] Khatib Z. A., Matsushime H., Valentine M., Shapiro D. N., Sherr C. J., Look A. T. (1993). Coamplification of the CDK4 gene with MDM2 and GLI in human sarcomas. *Cancer Research*.

[124] Reifenberger G., Reifenberger J., Ichimura K., Meltzer P. S., Collins V. P. (1994). Amplification of multiple genes from chromosomal region 12q13-14 in human malignant gliomas: preliminary mapping of the amplicons shows preferential involvement of CDK4, SAS, and MDM2. *Cancer Research*.

[125] Reifenberger G., Ichimura K., Reifenberger J., Elkahloun A. G., Meltzer P. S., Collins V. P. (1996). Refined mapping of 12q13-q15 amplicons in human malignant gliomas suggests CDK4/SAS and MDM2 as independent amplification targets. *Cancer Research*.

[126] Wikman H., Nymark P., Väyrynen A. (2005). CDK4 is a probable target gene in a novel amplicon at 12q13.3-q14.1 in lung cancer. *Genes Chromosomes and Cancer*.

[127] Muthusamy V., Hobbs C., Nogueira C. (2006). Amplification of CDK4 and MDM2 in malignant melanoma. *Genes Chromosomes and Cancer*.

[128] Kim H., Huang W., Jiang X., Pennicooke B., Park P. J., Johnson M. D. (2010). Integrative genome analysis reveals an oncomir/oncogene cluster regulating glioblastoma survivorship. *Proceedings of the National Academy of Sciences of the United States of America*.

